# Decitabine-Mediated Epigenetic Reprograming Enhances Anti-leukemia Efficacy of CD123-Targeted Chimeric Antigen Receptor T-Cells

**DOI:** 10.3389/fimmu.2020.01787

**Published:** 2020-08-18

**Authors:** Liangshun You, Qingmei Han, Li Zhu, Yijing Zhu, Changqian Bao, Chunmei Yang, Wen Lei, Wenbin Qian

**Affiliations:** ^1^Department of Hematology, College of Medicine, The First Affiliated Hospital, Zhejiang University, Hangzhou, China; ^2^Malignant Lymphoma Diagnosis and Therapy Center, College of Medicine, The First Affiliated Hospital, Zhejiang University, Hangzhou, China; ^3^Institute of Hematology, Zhejiang University, Hangzhou, China; ^4^Department of Hematology, College of Medicine, The Second Affiliated Hospital, Zhejiang University, Hangzhou, China

**Keywords:** decitabine, CAR-T immunotherapy, DNA-methylation, acute myeloid leukemia, T cell subsets, immune synapse

## Abstract

Chimeric antigen receptor (CAR) T cells represent a potentially curative therapy for patients with advanced hematological cancers; however, uncertainties surround the cell-intrinsic fitness as well as the exhaustion that restrict the capacity of CAR-T. Decitabine (DAC), a DNA demethylating agent, has been demonstrated to reverse exhaustion-associated DNA-methylation programs and to improve T cell responses against tumors. Here we show that DAC significantly enhances antileukemia functions of CD123 CAR-T cells *in vitro* and *in vivo*. Additionally, it inhibits the expression of DMNT3a and DNMT1. Using the Illumina Methylation EPIC BeadChip (850 K), we identified differentially methylated regions, most of which undergo hypomethylated changes. Transcriptomic profiling revealed that CD123 CAR-T cells treated with DAC were enriched in genes associated with naive, early memory T cells, as well as non-exhausted T cells. DAC treatment also results in upregulation of immune synapse-related genes. Finally, our data further suggest that DAC works through the regulation of cellular differentiation characterized by naive and memory phenotypes. Taken together, these findings demonstrate that DAC improves the anti-leukemia properties of CD123-directed CAR-T cells, and provides a basis for rational combinatorial CAR-T-based immunotherapy for patients with acute myeloid leukemia (AML).

## Introduction

Chimeric antigen receptor (CAR) T cells represent a potentially curative therapy for patients with advanced hematological malignancies. In recent multicenter clinical trials, CAR-T cells targeting the CD19 molecule have demonstrated high and durable response rates for patients with refractory or relapsed B-cell lymphoma (1, 2). Despite these promising results, about half of the patients treated with CD19 CAR-T cells do not achieve complete responses (CR), and a significant fraction of the patients achieving remission will subsequently relapse with either loss of the target surface antigen or poor ongoing persistence of CAR-T cells *in vivo* as result of their inefficient activation or even inhibition due to immunosuppressive tumor microenvironment (3–6), indicating the need for a strategy that can improve CAR-T therapy.

Recent studies suggest that intrinsic T cell defects may lead to dysfunction, decreased expansion, and poor persistence of CAR-T cells (7–9). It was known that DNA methyltransferase 3a (DNMT3a)-mediated *de novo* DNA methylation not only directly drives T cell suppression and exhaustion, but inhibits immune checkpoint blockade (ICB)-mediated rejuvenation of exhausted T cells (10). Moreover, methylation of DNA can also result in changes in T cell differentiation and activity by altering the transcription levels of a variety of immune-associated genes (10, 11). Decitabine (DAC), FDA-approved DNA demethylating agent, has been demonstrated to reverse exhaustion-associated DNA-methylation programs and to improve T cell responses against tumors (10). However, it is not known whether DAC can improve the efficacy of CAR-T cell therapy.

In this study, we investigated the effects of DAC on CD123 CAR-T cells, and studied the mechanisms of augmented antileukemia activity by DAC and CAR-T-based immunotherapy with attention to the alterations in DNA methylation, mRNA expression of immune-related genes, and T cell subsets.

## Results

### Decitabine Augments the Function of CD123 CAR-T Cells *in vitro*

**Figure 1:**
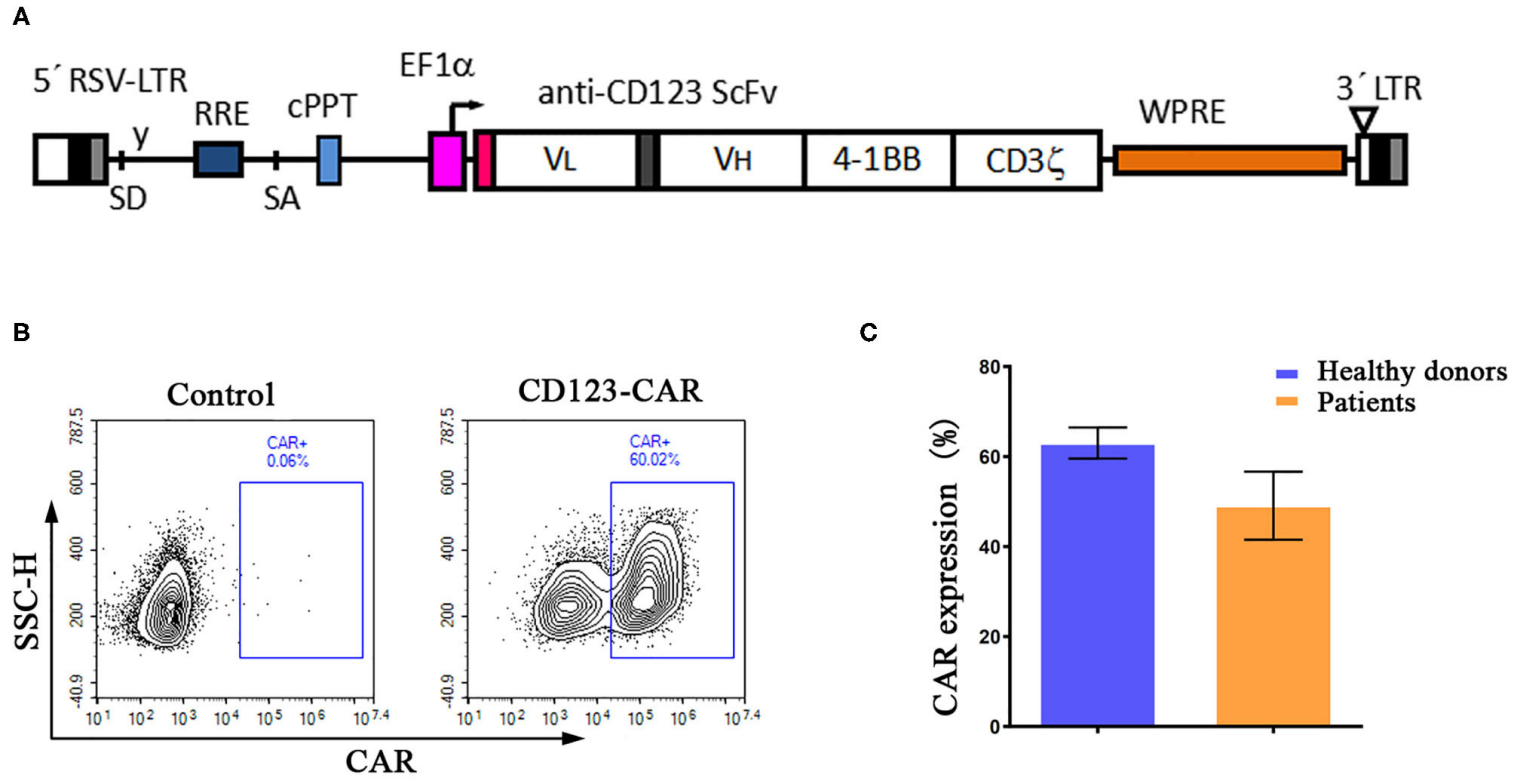
Human anti-CD123 specific T cells are generated by transducing chimeric CD123-CAR lentivirus. **(A)** Schematic representation of CD123-CAR structure. The CD123-CAR expression cassette is under the regulation of EF1α promoter, and mainly composed of an extracellular CD123-binding scFv, a 4-1BB costimulatory domain, and CD3ζ endodomains. **(B,C)** The transduction efficiency of T cells from patients (*n* = 2) and healthy donors (*n* = 3) was confirmed by fluorescence-activated cell sorting (FACS) analysis as described in Materials and Methods. Representative data of one healthy donor was presented **(B)**. The average expression (Mean ± SD) of CD123-CAR in transduced T cells from patients and healthy donors was shown respectively **(C)**.

**Figure 2:**
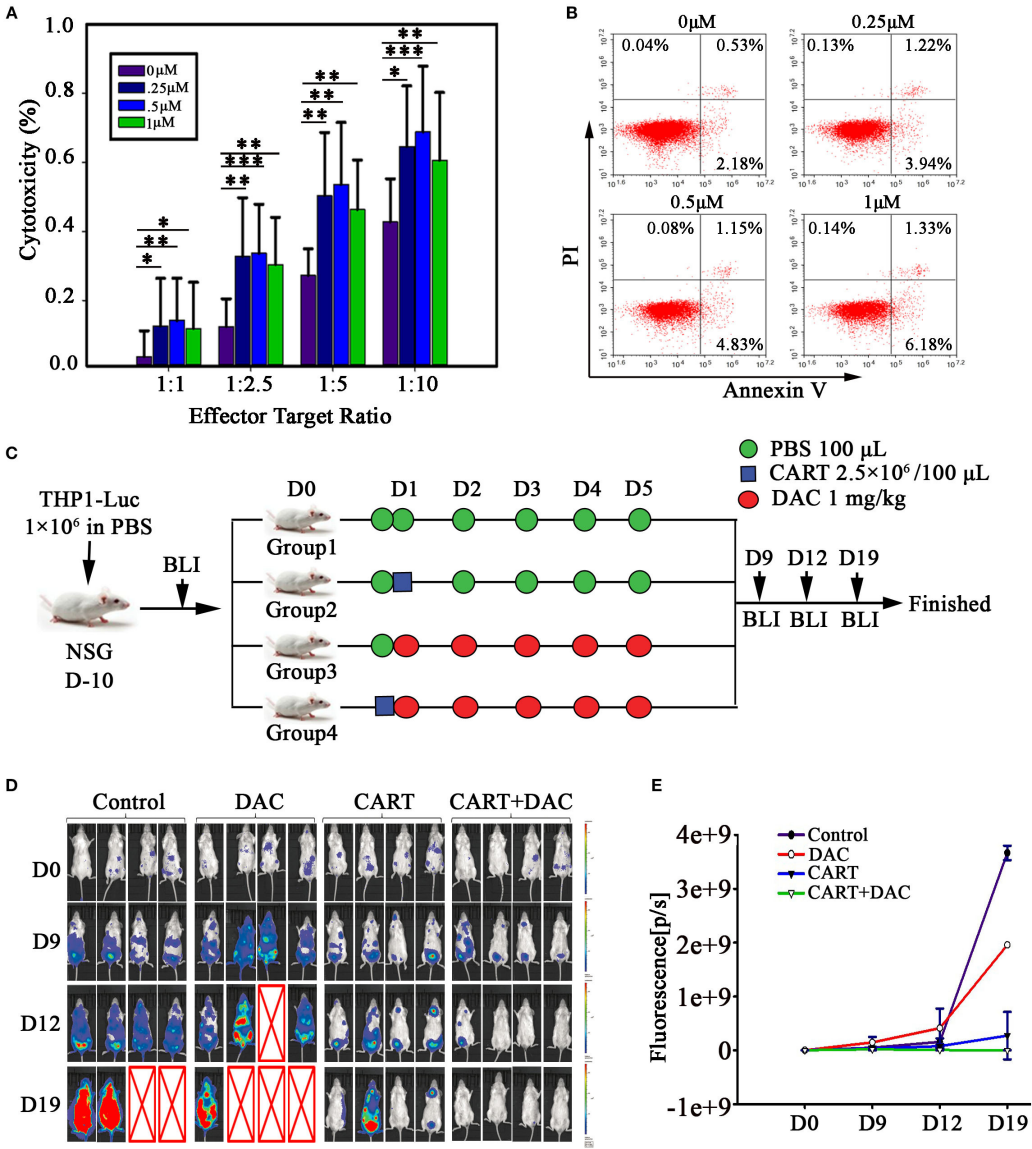
DAC enhances anti-leukemia activity of CD123 CAR-T cells *in vitro* and *in vivo*. **(A)** CD123 CAR-T cells, generated from four patients and three healthy donors, pretreated with different concentrations of DAC for 48 h, and then were co-cultured with THP1 cells at different E: T ratio (Effector: Target) for 4 h after drug wash-out. LDH release assay was used to detect the cytotoxicity of CD123 CAR-T cells. Three independent experiments were conducted. Mean ± SD. ^*^p < 0.05, ^**^p < 0.01, ^***^p < 0.001. **(B)** CD123 CAR-T cells were treated with different doses of DAC for 48 h and apoptosis was determined by flow cytometry. **(C)** NSG mice bearing AML tumor xenografts constructed by THP1-luciferase cells were randomized to receive one of the following treatments on 5 consecutive days: PBS, CD123 CAR-T cells (2.5 × 10^6^), DAC (1mg/kg), or CD123 CAR-T cells (2.5 × 10^6^) combined with DAC (1mg/kg). **(D)** Tumor signals were monitored with Lumina imaging on day 9, day12, and day 19. **(E)** The bioluminescence signal was measured as total photon flux normalized for exposure time and surface area and expressed in units of photons (p) per second per cm^2^ per steradian (sr) (*n* = 4 mice in each group).

It was reported that targeting DNA methylation impacts T cell function (12, 13). To study the effects of DAC treatment on anti-CD123 CAR-T cells, human peripheral CD3^+^ T cells were transduced with a lentiviral vector that encodes a CAR recognizing CD123 (Figure 1A). The CAR transduction efficiency was evaluated *via* FACS using anti-mouse F(ab′) 2-APC antibody (Figure 1B). Similar transduction efficiency were obtained in T cells from both healthy donor and the patient (Figure 1C). We next pretreated CD123 CAR-T cells with DAC at concentrations ranging from 0.25 to 1.0 μM and then carried out a wash to remove DAC in the culture medium. The DAC-pretreated CD123 CAR-T cells were co-cultured with THP1 cells for 48 h at different E: T and CAR-T cell killing was evaluated by LDH assay. As shown in Figure 2A, recommended low doses of DAC significantly enhanced activity of CAR-T cells. However, this enhanced cytotoxicity decreased when the CAR-T cells were pretreated with low doses (1 μM) of DAC.

### Impact of DAC on CD123 CAR-T Proliferation and Apoptosis

To investigate the effect of DAC on CAR-T cell proliferation, we assessed CAR-T cell proliferation through CFSE dilution. Pretreatment with DAC resulted in slightly decreased rate of proliferation in a dose-dependent manner when co-cultured with THP1 cells (Supplemental Figure 1). We next evaluated the possible toxic effect of DAC when treating CD123 CAR-T. DAC was added to CAR-T cell cultures at 0, 0.25, 0.5, and 1 μM for 48 h and slightly increased apoptosis was observed. However, when DAC was used at concentration (1 μM) similar to serum level detected in patients (14, 15), the apoptosis rate of CAR-T was only 7.68% (Figure 2B).

### Enhanced Antileukemia Activity of CD123 CAR-T Combined With DAC *in vivo*

Based on the positive results from our *in vitro* studies and on the evidence that DAC has been demonstrated to be effective in patients with AML (12, 13), we investigated *in vivo* antiproliferative effect of CD123 CAR-T and DAC. For this propose, we developed a xenograft model of THP1-luciferase cells in NSG mice that allowed us to use bioluminescence to measure tumor growth before and after the combination therapy. The schema of the treatment protocol using those agents is provided in Figure 2C. Mice were treated with DAC, CD123 CAR-T cells, or CAR-T cells combined with DAC. BLI demonstrated significantly reduced leukemia burden in combination group compared to CAR-T cells alone (Figures 2D,E). On day 19, leukemia elimination was observed in all mice with the combined treatment.

### DNA Methylation Landscape Between CD123 CAR-T and the CAR-T Cells Treated With DAC

**Figure 3:**
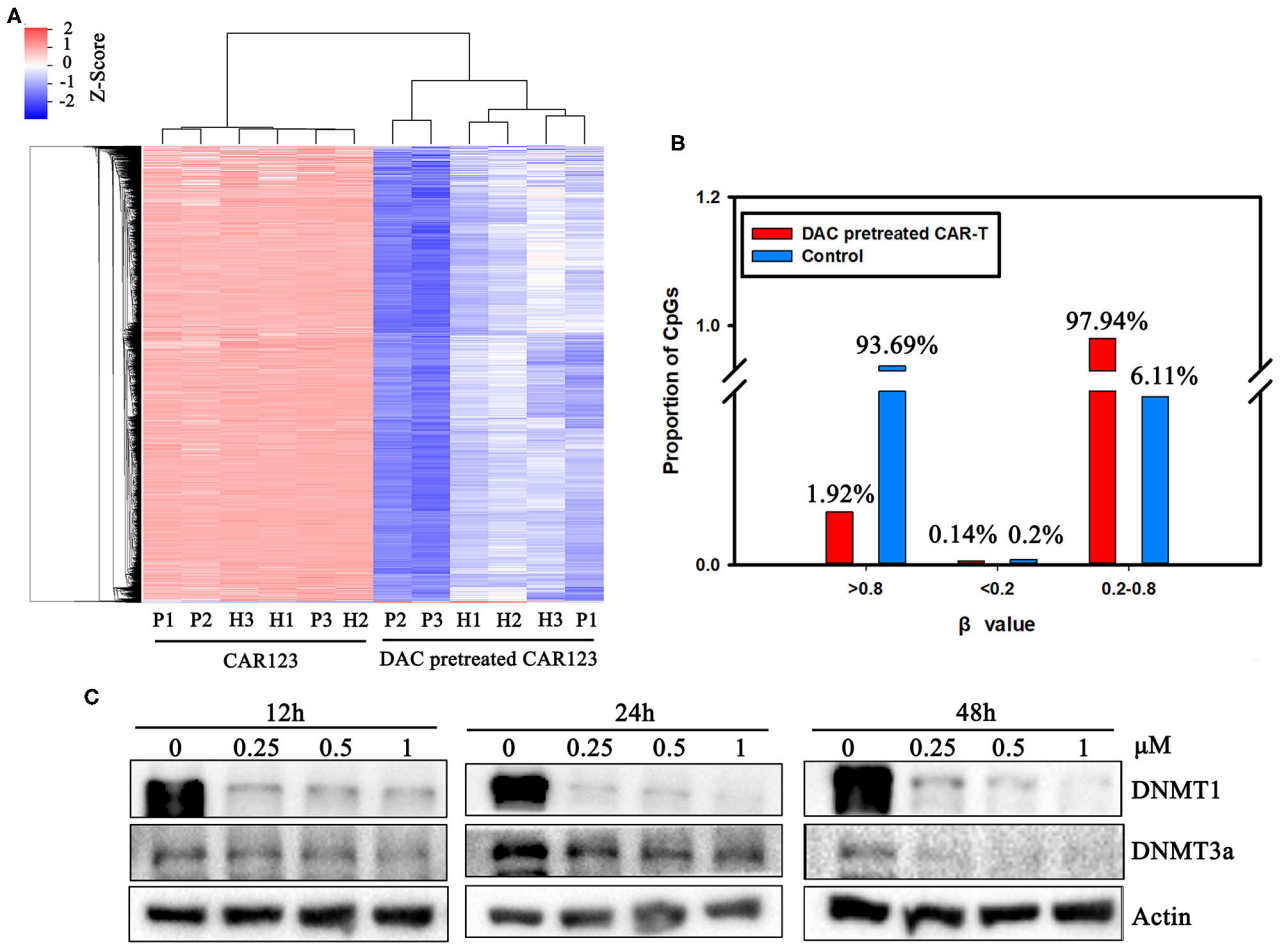
The divergence of DNA methylation landscape between CD123 CAR-T cells pretreated with and without DAC. **(A)** CD123 CAR-T cells from three patients (P1, P2, P3) and three healthy donors (H1, H2, H3) were treated with or without 1 μM DAC for 48 h. Illumina Infnium HD Methylation 850 K arrays were used to determine the DNA methylation status. Differential methylation CpG sites whose case Diffscore < −13 or >13 (*p* < 0.05) and case Delta_Beta >0.17 or < −0.17 were presented. **(B)** Percentage of high (β-values >0.8), medium (0.2 < β-values <0.8) and low (β-values <0.2) methylation level was presented with or without DAC pretreatment. **(C)** CD123 CAR-T cells from patients were treated with a series of doses of DAC for 12, 24, or 48 h. Whole-cell lysates were subjected to western blotting analysis to examine the protein levels of total DNMT1 and DNMT3a. β-actin was used as loading control. The data are representative of three determinations with identical results.

We next asked whether DNA methylation was altered in the CAR-T cells treated with DAC through genome-wide analysis of DNA methylation using the Illumina Infnium HD Methylation 850 K array. In this analysis, six matched pairs of DAC-treated and untreated CAR-T samples from both AML patients and healthy donors were included. Results showed that there were 4,957 differentially methylated CpG sites between CAR-T and DAC-treated CAR-T cells. We performed hierarchical clustering to explore whether DAC treatment resulted in differentially methylated regions (DMRs). As presented in the heatmap in Figure 3A, a functional dichotomy was observed, with CAR-T and CAR-T treated with DAC segregating into two distinct clusters. The CAR-T cells treated with DAC showed an obvious hypomethylated CpGs. Furthermore, methylation values in each group were measured as β-values. In DAC treated-group, 97.9% of CpGs had an intermediate methylation levels (β-values = 0.2–0.8). Whereas, in untreated-group 93.7% of CpGs had an extreme methylation with >0.8 β-value (Figure 3B).

The mechanism of action of DAC on CAR-T is as yet poorly understood. However, it is well-know that DNMT3a-mediated *de novo* DNA methylation programs promote T cell exhaustion (10) Therefore, we analyzed DAC-dependent alterations of the protein level of DNMT1 and DNMT3a, both of them are involved in DNA methylation and T cell development, function, and survival (11, 14). Western blotting showed that protein expressions of DNMT1 and DNMT3a were significantly downregulated in a dose- and time-dependent manner after drug treatment (Figure 3C, Supplemental Figure 2).

### Alterations of T Cell-Intrinsic Potency and Subsets May Contribute to an Enhanced CAR-T Cell Activity

**Figure 4:**
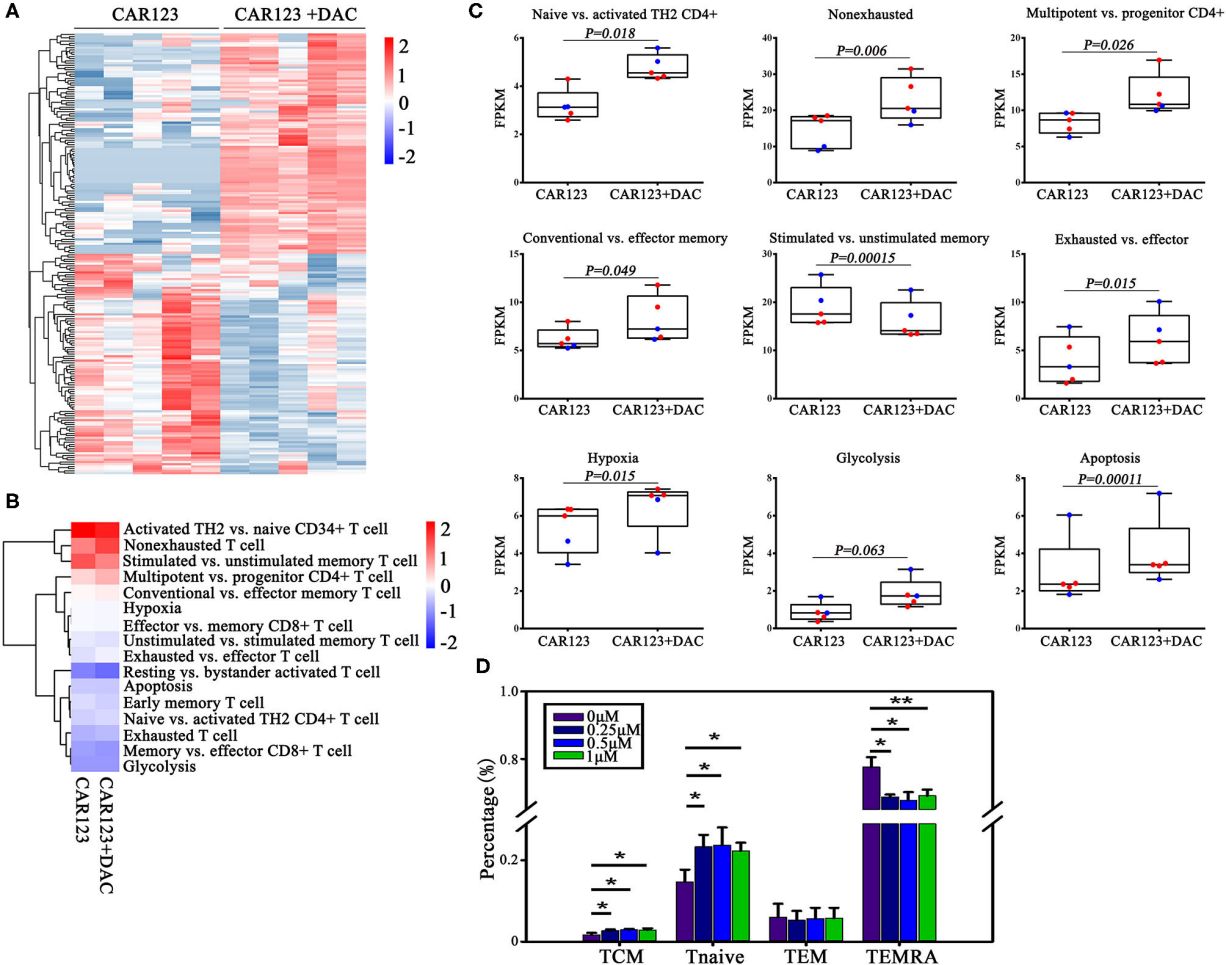
Investigations on the effect of DAC on intrinsic potency and subsets of CAR-T cells. CD123 CAR-T cells generated from two patients and three healthy donors were treated with or without 1 μM DAC for 48 h. Then RNA seq gene expression analysis was performed. **(A)** The top 200 genes in either direction were shown. **(B)** Heat map of T cell pathways enriched in genes upregulated or downregulated in DAC pretreated CD123 CAR-T cells. For each pathway, a single sample enrichment score was calculated by the expression level of FRKM of each gene (genes of each T cell subsets detailed in Supplementary Table 2), and the mean was taken by group. Each column represented an individual sample pretreated with or without DAC, and each row represented the enrichment of gene expression profiles involved in different subsets of T cells. A color gradient represents the changes of mean normalized enrichment score from red (highest) to blue (lowest) (ranging from −2 to +2). Blue indicates a down-regulation and Red indicates an up-regulation. **(C)** Enrichment of T cell pathways in CD123 CAR-T cells were presented. Each point represents a specimen. Red for patients and green for healthy donors. Mean ± SD. **(D)** CD123 CAR-T cells (*n* = 3) were treated with a series of doses of DAC for 48 h with the presence of THP1 at E:T = 1:1. T cell subsets (Tcm: CD45RA–CCR7+; Tnaive: CD45RA+CCR7+; Tem: CD45RA–CCR7–; T_EMRA_: CD45RA+CCR7–) were measured by FACS analysis. Three independent experiments were conducted. Mean ± SD. ^*^p < 0.05, ^**^p < 0.01.

To understand the relationship between DNA methylation change and gene expression regulation, we performed RNA seq analysis to compare the difference of their transcriptional profiles in the CAR-T cells that treated with or without DAC. Thousand six hundred forty two genes were found to be differentially expressed (fold change >1.2) in the RNA-seq experiment, among them 1,091 genes were up-regulated and 551 genes were down-regulated in CD123 CAR-T cells treated with DAC (Figure 4A, full gene names are listed in Supplemental Table 1). Then we analyzed differential expression of genes that are associated with T cell function and subsets (8, 15). We subdivided these genes into sixteen specific pathways (Figure 4B, detailed gene names for each T cell subtypes are listed in Supplemental Table 2). The heatmap showed that DAC treated-CAR-T cells were enriched in gene expression profiles involved in naive T cells, multipotent, effector memory, and non-exhausted T cells (Figure 4C). Next, we analyzed negative correlations between the expression of genes involved in T cell function and subsets and DNA methylation data, and found that upregulation of SLC12A7 and MAST3 with promoter hypomethylation (Supplemental Table 3). It has been reported that CAR-T cells from chronic lymphocytic leukemia (CLL) patients who achieved CR/partial remission expressed key regulators of cellular memory differentiation and maintenance (8). We thus examined the effects of DAC on subset of CAR-T cells. The CAR-T cells treated with DAC showed less differentiated phenotypes based on CD45RA and CCR7 expression, with higher proportions of CAR-T cells with naive and T_CM_ cell phenotypes. Whereas, the CAR-T untreated with DAC showed a more differentiated phenotype with a higher proportion of cells with T_EMRA_ cell phenotype (Figure 4D).

### Decitabine Enhances Transcriptional Signatures of Immune Synapse

**Figure 5:**
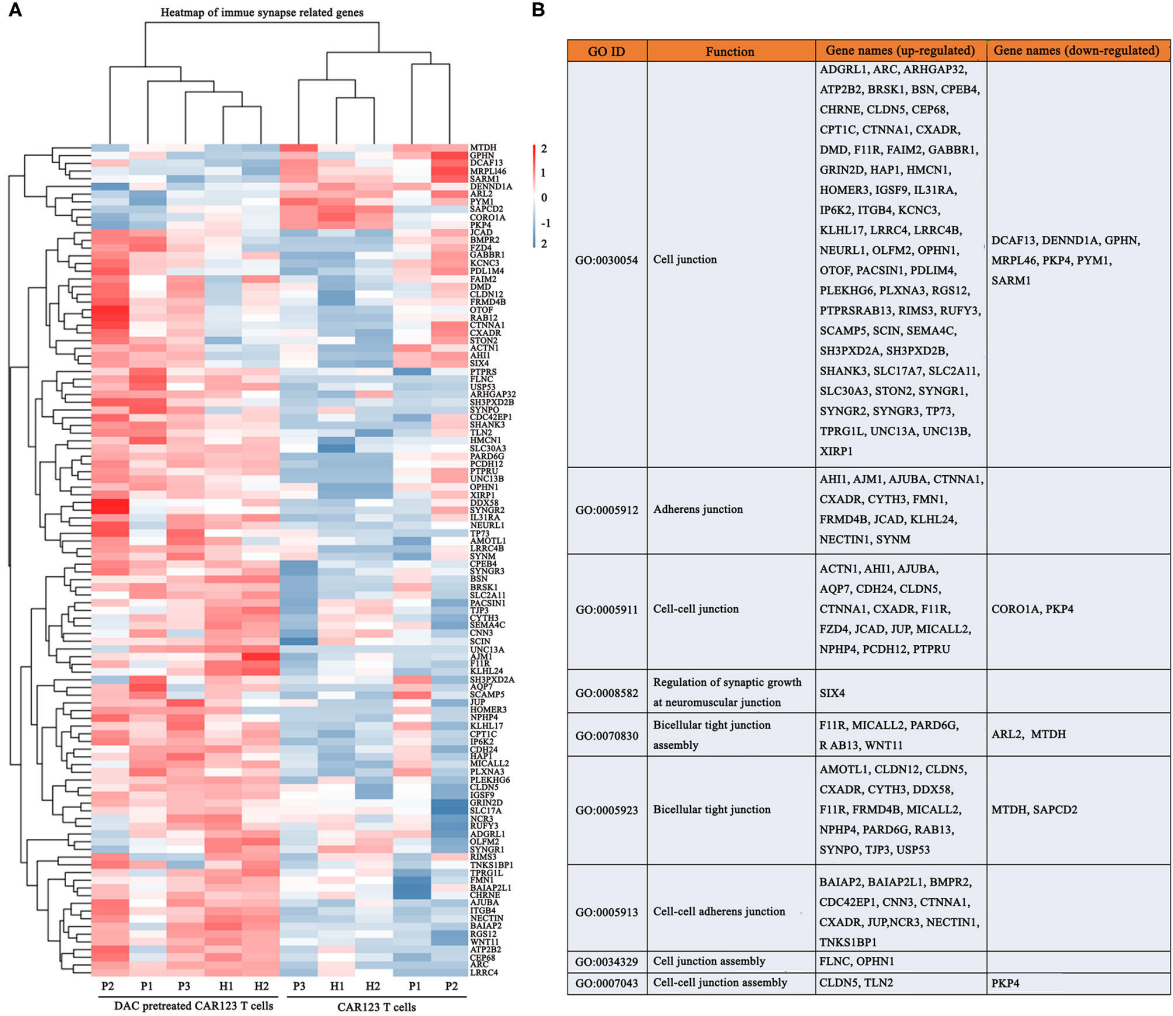
DAC enhances transcriptional signatures of immune synapse in CD123 CAR-T cells. The information of CD123 CAR-T cells and RNA seq gene expression analysis were the same as the Figure 5. **(A)** Immune synapse genes were presented as heat map. Each column represented an individual sample pretreated with or without DAC, and each row represented an individual gene. **(B)** Gene ontology (GO) enrichment of these immune synapse genes.

It is known that CAR-T cells utilize non-classical and, at least in part, the classical immune synapse, which are required for their effector function (16, 17). We therefore, analyzed whether DAC induce different transcriptional changes of immune synapse-related genes. Gene-expression analyses of CAR-T cells revealed significant transcriptional responses induced by DAC. Ninety-seven genes were upregualted, and 11 genes were downregulated (Figure 5A). These up-regulated genes were mainly associated with immune synapse-related functions, including GO categories such as cell junction (GO: 0005912), adherens junction (GO: 0005911), and bicellular tight junction (GO: 0005923) (Figure 5B, Supplemental Table 4). We also analyzed the methylation status of these genes. There were three genes that showed a significant increase in gene expression and decrease in promoter DNA methylation: FLNC, SEMA4C, and MICALL2 (Supplemental Table 5).

## Discussion

CAR-T cell therapy represents an effective and durable therapy for patients with refractory/relapsed B cell malignancies. However, resistance to CAR-based immunotherapy is frequently observed (4–6), suggesting that it is necessary to develop novel strategies to enhance CAR-T cell efficacy. In this study, we evaluated a novel combination therapy for AML and explored the underlying mechanisms. The approach uses a demethylating agent, DAC, in combination with CD123 CAR-T cells. It has been proved that DAC not only can inhibit DNA methylation in low dose, but also can induce cell apoptosis in high dose (18, 19). To avoid inhibiting T cell activity, we adopted the recommended low-dose DAC (0.1–1 μM) in our experiment (18, 19). We demonstrated for the first time that pretreatment with DAC augments activation of CD123 CAR-T cells *in vitro* and also show an enhanced antileukemia effect in the context of combination treatment *in vivo*. Consistent with these results, Li et al. (20) reported that lymphoma cells were more sensitive to killing by CD19 CAR-T cells following pretreatment with DAC, and that two patients with refractory/relapsed B-cell lymphoma received sequential therapy (DAC followed by CAR-T cells), of whom both achieved CR. Together, these data may indicate the effectiveness of combinational treatment with DAC and CAR-T cells on patients with AML.

T cells can be rendered dysfunctional by both intrinsic and extrinsic mechanisms (5, 6, 21). Ghoneim et al. (10) demonstrated that *de novo* methylation-mediated by DNMT3a in an effector CD8^+^ T cell response to a tumor challenge is critical for establishing T cell exhaustion and that DAC may function by reversing exhaustion-associated *de novo* methylation programs in CD8^+^ T cells, therefore enhancing the T cell expansion during ICB treatment. Moreover, deletion of DNMT3a at an early stage of effector differentiation inhibited methylation of naive-associated genes and induced re-expression of these naive genes and the resultant memory cell development (22), which may induce potent anti-cancer responses in adoptive cell therapy (9). Here, we show that DAC treatment significantly inhibits the expression of DMNT3a and DNMT1, and also plays an important role in T cell development, function, and survival (14, 23), in CAR-T cells. We next focused on genome-wide differences in DNA methylation between the DAC- and mock-treated CAR-T cells because this modification has been shown to be involved in CD8^+^ T cell differentiation (24). In subjects exposed to DAC, we observed lower methylation levels in CpG islands (CGIs), CGI shores and shelves in which its methylation also affects gene expression (24). Next, we performed a gene ontology analysis to address the functional significance of the wide-spread changes in DNA methylation, which revealed that the hypomethylated DMRs identified multiple biological processes, including ATP binding, protein serine/threonine kinase activity, GTPase activator activity, etc. (Supplemental Figure 3). To our knowledge, this is the first study to show that DAC modifies the DNA methylation landscape in the CD123 CAR-T cells.

It is well-known that naive and memory T cells are superior to effector T cells in induction of potent anti-tumor responses for adoptive cell therapy (9). A recent landmark clinical study compared the transcriptomes of CAR-T cells generated from CLL patients who respond well or do not respond to CD19 CAR-T therapy, and confirmed that CAR-T cells from responder expressed key regulators of early memory differentiation while CAR-T cells from non-responder exhibited elevated expression of key regulators of effector T cell differentiation and exhaustion, suggesting these factors are associated with therapeutic efficacy. Moreover, the genes significantly upregulated in unmanipulated T cells from non-responders were enriched in pathways that regulate the exhaustion, activation, glycolysis and apoptosis (8). In the present study, immune cell phenotyping shows that DAC-treated CAR-T cells had a significantly higher percentage of naive and T_CM_ phenotypes and a lower frequency of T_EMRA_ phenotype. Consistent with this, transcriptomic profiles of DAC-treated CAR-T cells showed marked differences compared with those from untreated group. The genes significantly upregulated in DAC-treated CAR-T cells were enriched in the key transcriptional regulators that are associated with naive, effector memory, unstimulated memory subset, and non-exhausted T cells. These findings revealed that DAC may function by reversing exhaustion-associated DNA-methylation programs in CAR-T cells and upregulating the expression of genes that favor naive and memory T cells differentiation, which may contribute to an enhanced CAR-T cell function.

The non-classical immune synapse is also key factor for determining the rapid cytotoxicity of CAR-T cells (17, 25, 26). Recently, Wang et al. (27) reported that lenalidomide induced upregulation of immune synapse-related genes such as cell junction and biological assembly-related genes, ultimately leading to the enhanced immune synapse formation, which is associated with the improved cytolytic activity of CS1 CAR-T cells against myeloma. We thus analyzed the effects of DAC on immune synapse-related genes. The results showed that significant DAC-related changes in expression were observed for 108 genes. Of these genes, 97 were higher expressed and 11 were lower expressed with DAC treatment. These data provided the clue that modulation of immune synapse might be a mechanism of action of DAC, although more research will be needed in future concerning these issues.

## Conclusion

In summary, this is the first preclinical study that provides novel mechanistic insights into CAR-T cells functional augmentation by DAC. These findings suggest that methylation modulation might be a strategy to enhance anti-tumor immunity for adoptive CAR-T cell therapy, which paves the way for clinical trials using the combination of methylation inhibitors with engineered T cells against leukemia as well as other cancers.

## Materials and Methods

### Cell Line, Human Peripheral Blood Samples, and Reagents

The human acute myeloid leukemia (AML) cell line THP1 were purchased from the American Type Culture Collection (ATCC; Manassas, VA, USA) and cultured as described previously (28). Peripheral blood samples were obtained from healthy donors (*n* = 4) and AML patients (*n* = 4), respectively, in The first Affiliated Hospital, Zhejiang University after informed consent according to the institutional guidelines. The peripheral blood mononuclear cells (PBMNCs) were enriched through a Ficoll Hypaque gradient. Decitabine (DAC, 5-Aza-2′-deoxycytidine) was supplied as a powder (Sigma-Aldrich, St. Louis, USA), and dissolved in DMSO at a concentration of 50 mg/ml before use.

### Generation of CD123 CAR-T Cells

The CD123-CAR lentivirus was kindly provided by Professor Gao Jimin from Wenzhou Medical University, China. The CAR contained an extracellular single chain variable fragment (scFv) specific for CD123 and multiple costimulatory domains including 4-1BB and CD3ζ. Purified CD3^+^ cells from human PBMNCs were activated with anti-CD3 and anti-CD28 dynabeads (Gibco, Grand Island, NY, USA). Next, lentivirus encoding CD123-CAR was transduced into activated CD3^+^ T cells in the presence of IL-2, IL-7, and IL-15. After transduction, T cells continued to be cultured in X-VIVO medium (Gibco) containing 10% heat-inactivated fetal bovine serum (FBS, Gibco), 300 IU/ml IL-2, 5 ng/mL IL-7, and IL-15 (Prime Gene, Shanghai, China) in a 37°C, 5% CO_2_ humidified incubator.

### Antibodies and Flow Cytometry Analysis

Fluorochrome-conjugated isotype against human CD3 (APC/Cy7/FITC, UCHT1), CD4 (Pacific Blue, OKT4), CD8 (PE, SK1, 344706), CD45RA (PE/Cy7, HI100), CCR7(APC, G043H7), CD123 (FITC, 6H6), and streptavidin-Alexa Fluor 594 were purchased from Biolegend (San Diego, CA, USA). The antibody of Biotin-SP-conjugated AffiniPure Goat Anti-Mouse IgG, F(ab′)2 Fragment Specific was obtained from Jackson Immunoresearch. Samples were analyzed using the ACEA NovoCyte flow cytometer and the Novoexpress software (ACEABIO). For detection of CAR surface expression, T cells were incubated with Biotin-conjugated Anti-Mouse F(ab′)2 for 20 min and followed by two washes and stained with streptavidin-Alexa Fluor 594 and FITC labeled CD3 for another 20 min at 4°C. Based on the phenotype and functions, T cells would be divided into four different subsets: naïve T cell (T_naive_, CCR7^+^ CD45RA^+^), central memory T cell (T_CM_, CCR7^+^ CD45RA^−^), effector memory T cell (T_EM_, CCR7^−^CD45RA^−^) and CD45RA^+^ effector memory T cell (T_EMRA_, CCR7^−^CD45RA^+^).

### Cytotoxicity Assay

Cytotoxic activity of CD123 CAR-T cells were evaluated by a cytotoxicity detection kit (Promega, Madison, WI, USA) according to the manufacturer's instructions. The indicator for cytotoxicity was the amount of lactate dehydrogenase (LDH) released from targeted cells. In brief, THP1 cells (1 × 10^5^/ml) were plated into the 96-well culture plate in triplicate following by adding CAR-T cells at the indicated effector to target ratios (E: T) ratios and then co-incubated for 4 h. The supernatants were collected and analyzed for LDH activity.

### Western Blot Assay

CAR-T cells were treated with various concentrations of DAC for different times. Total proteins were extracted and protein concentration was detected by BCA Protein Assay Kit (Sangon Biotech, Hangzhou, China) according to the manufacturer's manual. An equal amount of total proteins was subjected to Western blotting as previously described (28). The membranes were visualized using the ECL kit (Biological Industries, Beit Ahemeq, Israel) and images were obtained by the ChemiDoc MP Imaging System (Bio-Rad, Hercules, USA). The primary antibodies used in this study included anti-DNMT1, anti-DNMT3A antibodies, and anti-β-actin was used to ensure equivalent loading of whole-cell protein, which were purchased from Cell Signaling Technology (Beverly, MA, USA).

### CAR-T Cells Proliferation Assay

CAR-T cells, with or without DAC treatment, were stained with 1 μM carboxyfluorescein diacetate succinimidylester (CFSE, Invitrogen, Waltham, MA, USA) for 15 min at 37°C in dark. The reaction was terminated with an equal volume of pre-warmed X-VIVO containing 10% heat-inactivated FBS (Gibco) for another 5 min at 37°C. After washed twice with PBS, CFSE stained CAR-T cells were incubated at a 1:1 ratio with target cells for 72 h and subsequently analyzed by FACS.

### Genome-Wide Analysis of DNA Methylation

Illumina Infnium HD Methylation 850 K arrays were used to determine the DNA methylation status of 850,000 CpG sites, of which 350,000 CpG sites for detection of enhancers, according to the manufacturer instructions. Infnium Human Methylation 850 BeadChip includes 851,764 cytosine positions of the human genome covering >14,000 genes. The DNA methylation levels for each CpG site were computed as the ratio of normalized methylated signal intensity to the sum of methylated and unmethylated signal intensities using GenomeStudio software, showed as the β-values which range from 0 to 1, corresponding to completely unmethylated and fully methylated sites, respectively. When the case Diffscore < −13 or >13 (*P* < 0.05) and the case Delta_Beta >0.17 or < −0.17 using *T*-Test Model, we identified the sites as the differential methylation CpG sites.

### RNA Sequencing (RNA-Seq) Analysis of Transcriptional Profile

Total RNA from CAR-T cells were extracted using the mirVana miRNA Isolation Kit (Ambion, USA). RNA integrity was evaluated using the Agilent 2,100 Bioanalyzer (Agilent Technologies, Santa Clara, CA, USA). The samples with RNA Integrity Number (RIN) ≥7 were subjected to the subsequent analysis. The libraries were constructed using TruSeq Stranded mRNA LTSample Prep Kit (Illumina, San Diego, CA, USA). Then these libraries were sequenced on the Illumina HiSeqTM 2,500 platform and 125 bp/150 bp paired-end reads were generated. The transcriptome sequencing and analysis were conducted by OE biotech Co., Ltd. (Shanghai, China). *P* < 0.05 and fold-Change >1.2 or fold-Change <0.83 were set as the threshold for significantly differential expression. Hierarchical cluster analysis of DEGs was performed to explore genes expression pattern.

### *In vivo* Leukemia Xenograft Study

The animal experiments was approved by the Institutional Animal Care and Use Committee, Zhejiang University. Six-week old female NOD.Cg-Prkdc^scid^ IL2rg^tm1Wjl^/SzJ (NSG) mice were purchased from Biocytogen (Beijing, China). Luciferase-expressing THP1 cells (1 × 10^6^) were inoculated intravenously (i.v) to construct AML model. After successful leukemia engraftment that was confirmed by a non-invasive *in vivo* bioluminescent imaging (BLI) system, mice were randomized to three treatment groups (*n* = 4), given CD123 CAR-T cells (2.5 × 10^6^) with or without DAC (1 mg/kg for 5 days), DAC (1 mg/kg for 5 days) and the control group (*n* = 4). In these studies, all test CAR-T cells and DAC were administered by i.v. route. Anesthetized mice were imaged using IVIS^®^ Lumina LT instrument (PerkinElmer, Walthan, Massachusetts, USA) on day 9, day 12, and day 19 to observe changes in tumor burden. Photons from Luc^+^ tumor xenografts were quantified using the software program Living Image (PerkinElmer).

### Statistical Analysis

Analyses were performed with SPSS 24.0. The paired *t*-test or Wilcoxon signed rank test were used to assess the statistical significance of the *in vivo* data. The paired *t*-test were used for the analysis of *in vitro* data. Negative binomial regression was used for the analysis of RNA-Seq to identify different gene expression. All tests were two sided. The *P*-value, below 0.05 was considered as statistically significant. All data were presented as mean ± standard deviation.

## Data Availability Statement

The sequencing data can be found on NCBI—PRJNA607611. Other raw data supporting the conclusions of this article will be made available by the authors, without undue reservation, to any qualified researcher.

## Ethics Statement

The animal experiments was approved by the Institutional Animal Care and Use Committee, Zhejiang University. The studies involving human participants were reviewed and approved by an independent Ethics Committee of The First Affiliated Hospital, College of Medicine, Zhejiang University. The patients/participants provided their written informed consent to participate in this study.

## Author Contributions

WQ designed the study. QH, LY, LZ, YZ, CY, and CB performed research and analyzed data. LY, WQ, and QH wrote the paper. All authors participated in the drafting of the manuscript and approved its final.

## Conflict of Interest

The authors declare that the research was conducted in the absence of any commercial or financial relationships that could be construed as a potential conflict of interest.

## Supplementary Material

Supplemental Figure 1 The effect of DAC on the proliferation of CAR-T cells. The proliferation of CD123 CAR-T cells was assessed using a Carboxyfluorescein diacetate succinimidyl ester (CFSE) assay, followed by flow cytometry analysis. CD123 CAR-T cells generated from patients (*n* = 4) **(A)** and health donors (*n* = 4) **(B)**.

Supplemental Figure 2 The difference in the level of protein expression was semi-quantitatively determined by densitometry and expressed as a ratio.

Supplemental Figure 3 GO and KEGG pathway analysis of differential methylation in RefGenes promoter regions. **(A–C)** were the top 20 RefGenes with greatest difference in the GO enrichment of biological process, molecular function, and cellular component respectively. **(D)** was the top 20 RefGenes with greatest difference in KEGG pathways.

Supplemental Table 1 The detail of RNA-Seq analysis of different expressed genes. Blue font indicates increased expression genes in CAR T cells after incubated with DAC and red font indicates decreased expression genes.

Supplemental Table 2 The genes of specific pathways for each T cell subtype. Blue font indicates increased expression genes in CAR-T cells after incubated with DAC and red font indicates decreased expression genes.

Supplemental Table 3 The relationship analysis of methylation and RNA Seq for specific pathways in CAR-T cells.

Supplemental Table 4 The expression of immune synapse-related genes. Blue font indicates increased expression genes in CAR-T cells after incubated with DAC and red font indicates decreased expression genes.

Supplemental Table 5 The relationship analysis of methylation and RNA Seq for synapse-related genes.

## Abbreviations

AML: acute myeloid leukemia
ATCC: American Type Culture Collection
BLI: bioluminescent imaging
CAR: Chimeric antigen receptor
CFSE: carboxyfluorescein diacetate succinimidylester
CR: complete response
CLL: chronic lymphocytic leukemia
DAC: Decitabine
LDH: lactate dehydrogenase
PBMNCs: peripheral blood mononuclear cells
RNA-Seq: RNA sequencing
TCM: central memory T cell
TEM: effector memory T cell
TEMRA: effector memory T cell
Tnaive: naïve T cell.
