# Supplementary figures and images for: Decitabine-Mediated Epigenetic Reprograming Enhances Anti-leukemia Efficacy of CD123-Targeted Chimeric Antigen Receptor T-Cells
Source: Front Immunol. 2020 Aug 18;11:1787. doi: 10.3389/fimmu.2020.01787 (PMC7461863; doi:10.3389/fimmu.2020.01787)

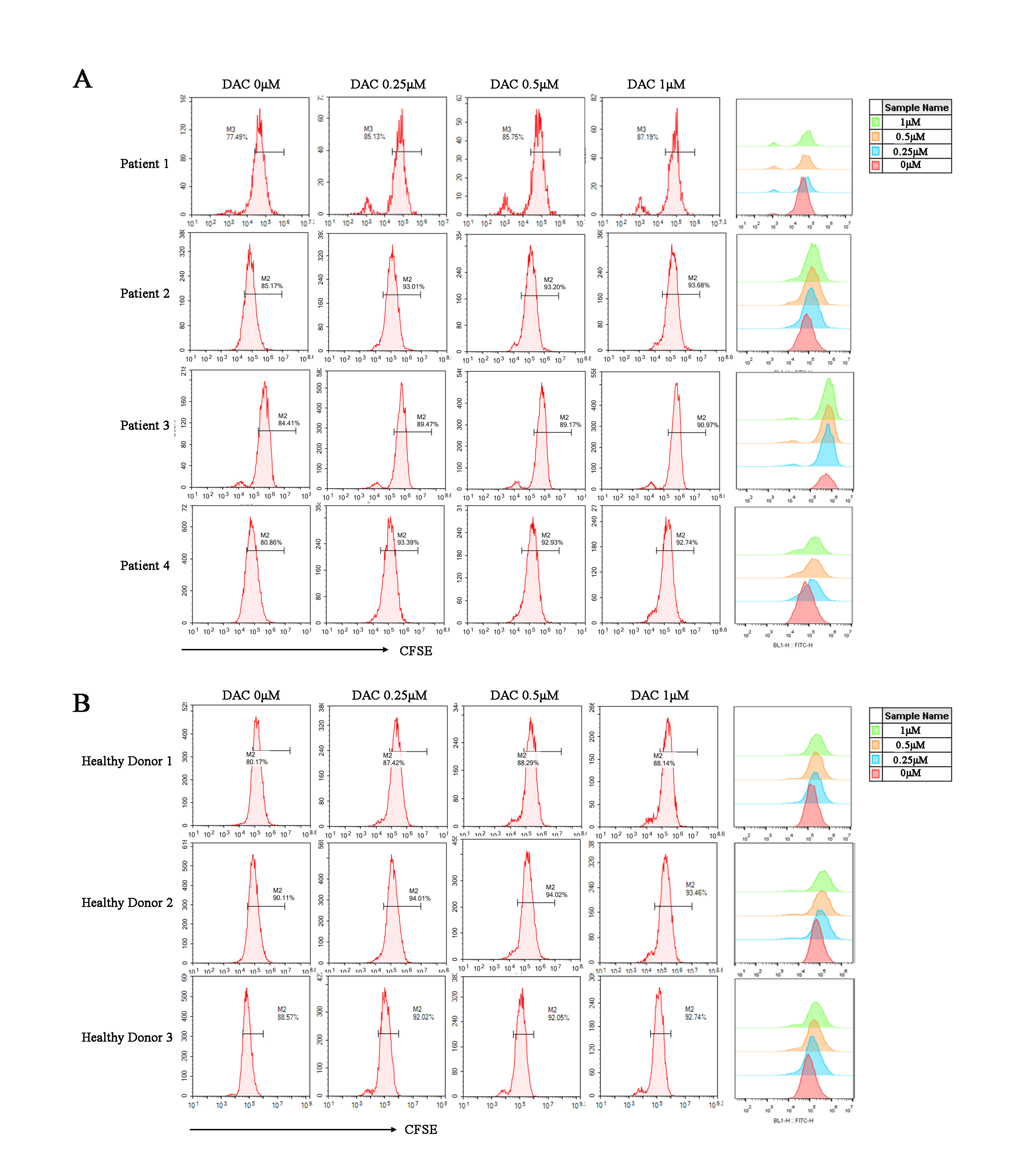

Supplement: Supplemental Figure 1 — The effect of DAC on the proliferation of CAR-T cells. The proliferation of CD123 CAR-T cells was assessed using a Carboxyfluorescein diacetate succinimidyl ester (CFSE) assay, followed by flow cytometry analysis. CD123 CAR-T cells generated from patients (n = 4) (A) and health donors (n = 4) (B). [file Image_1.tif]

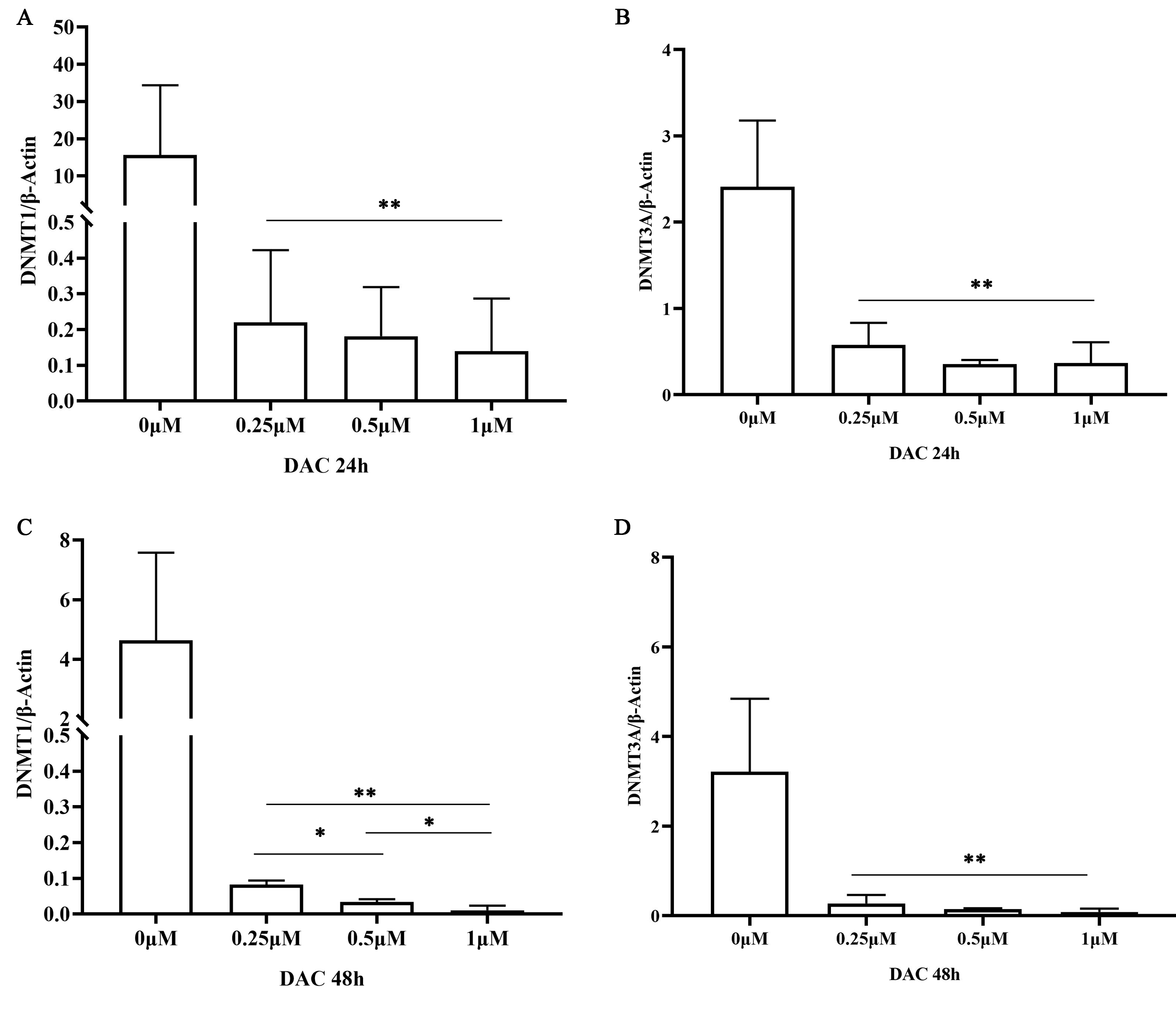

Supplement: Supplemental Figure 2 — The difference in the level of protein expression was semi-quantitatively determined by densitometry and expressed as a ratio. [file Image_2.tif]

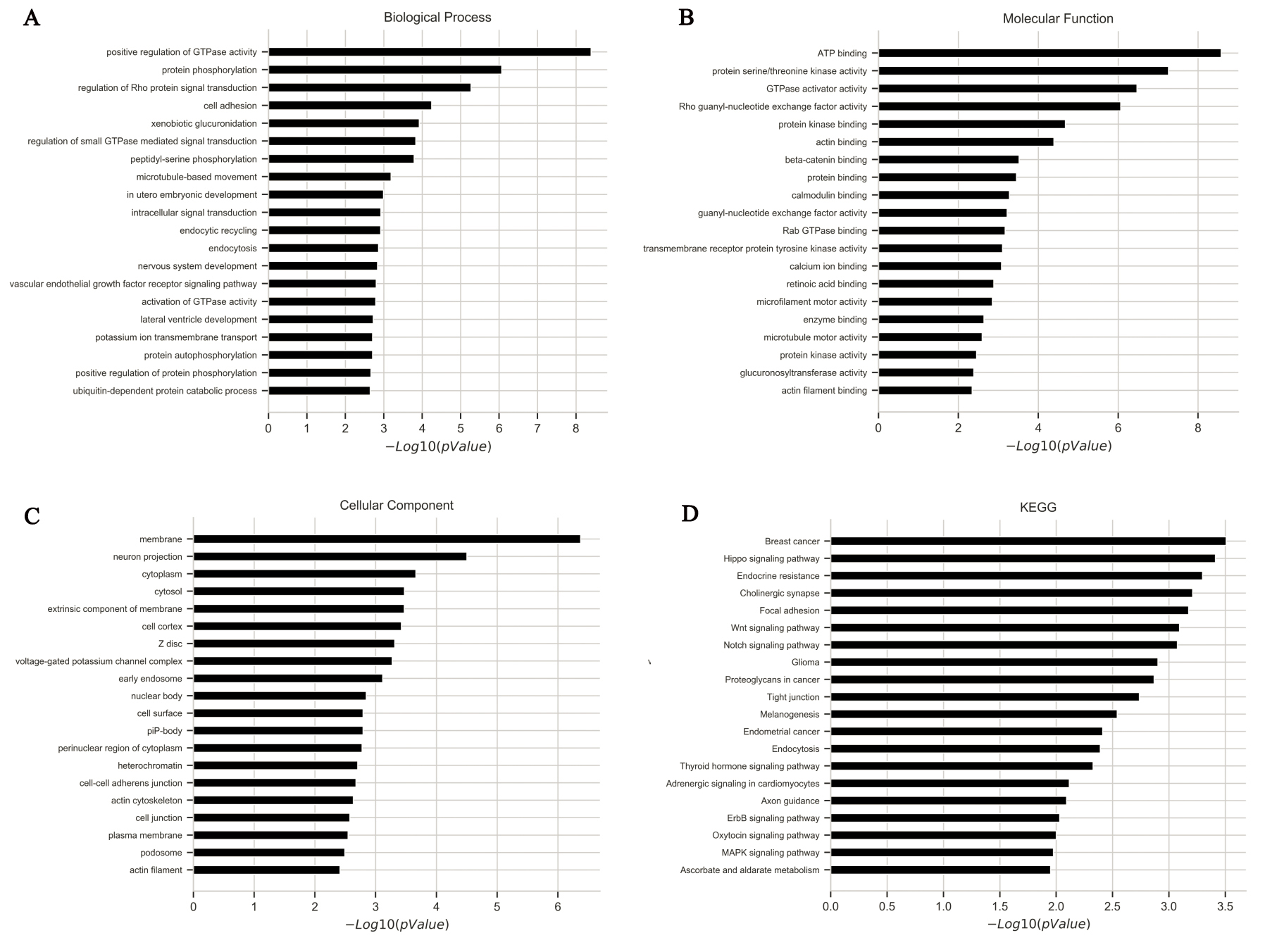

Supplement: Supplemental Figure 3 — GO and KEGG pathway analysis of differential methylation in RefGenes promoter regions. (A–C) were the top 20 RefGenes with greatest difference in the GO enrichment of biological process, molecular function, and cellular component respectively. (D) was the top 20 RefGenes with greatest difference in KEGG pathways. [file Image_3.tif]
